# Proteomic Analysis of Spore Surface Proteins and Characteristics of a Novel Spore Wall Protein and Biomarker, EhSWP3, from the Shrimp Microsporidium *Enterocytozoon hepatopenaei* (EHP)

**DOI:** 10.3390/microorganisms10020367

**Published:** 2022-02-04

**Authors:** Xiaodong Fan, Chunmei Wei, Xiaojuan Yang, Ai Xiao, Nianqiu Tan, Jie Chen, Mengxian Long, Guoqing Pan, Yongji Wan, Zeyang Zhou

**Affiliations:** 1 Laboratory of Invertebrate Pathology and Applied Microbiology, College of Sericulture, Textile and Biomass Sciences, Southwest University, Chongqing 400715, China; fanxd@cqnu.edu.cn; 2 Chongqing Key Laboratory of Microsporidia Infection and Control, State Key Laboratory of Silkworm Genome Biology, Southwest University, Chongqing 400715, China; jchen@swu.edu.cn (J.C.); longmx@swu.edu.cn (M.L.); zyzhou@cqnu.edu.cn (Z.Z.); 3 Chongqing Key Laboratory of Animal Biology, College of Life Sciences, Chongqing Normal University, Chongqing 401331, China; 2020110513024@stu.cqnu.edu.cn (C.W.); wei19331@163.com (X.Y.); 2020210513060@stu.cqnu.edu.cn (A.X.); 2021110513035@stu.cqnu.edu.cn (N.T.)

**Keywords:** microsporidia, *Enterocytozoon hepatopenaei*, spore wall protein, high-abundance surface proteins (HASPs), polyclonal antibodies

## Abstract

*Enterocytozoon hepatopenaei*, a spore-forming and obligate intracellular microsporidium, mainly infects shrimp and results in growth retardation and body length variation, causing huge economic losses to the Asian shrimp aquaculture industry. However, the lack of a full understanding of the surface proteins of spores associated with host infection has hindered the development of technologies for the detection of EHP. In this study, the surface proteins of EHP spores were extracted using the improved SDS method, and 130 proteins were identified via LC-MS/MS analysis. Bioinformatic analysis revealed that these proteins were enriched in biological processes (67), cellular components (62), and molecular functions (71) based on GO terms. KEGG pathway analysis showed that 20 pathways, including the proteasome (eight proteins) and the fatty acid metabolism (15 proteins), were enriched. Among 15 high-abundance surface proteins (HASPs), EhSWP3 was identified as a novel spore wall protein (SWP), and was localized on the endospore of the EHP spores with an indirect immunofluorescence and immunoelectron microscopy assay. Polyclonal antibodies against EhSWP3 showed strong species specificity and high sensitivity to the hepatopancreas of EHP-infected shrimp. As a specific high-abundance protein, EhSWP3 is therefore a promising target for the development of immunoassay tools for EHP detection, and may play a crucial role in the invasion of EHP into the host.

## 1. Introduction

Microsporidia, unicellular obligate intracellular spore-forming eukaryotic parasites, were previously regarded as the earliest-diverging clade of fungi [1], and recently were thought to be part of an independent lineage group that included Cryptomycota, Rozella, and Chytridiopsida, possessing many remarkable structural and functional differences from any other organisms [2,3,4]. Currently, at least 1400 species (200 genera) of microsporidia have been identified that can infect a variety of hosts, from invertebrates to vertebrates. Among them, more than 50 genera can infect aquatic arthropods, and these are considered to be the most important and common pathogens of aquatic arthropods [5]. Shrimps infected with microsporidia *Enterocytozoon hepatopenaei* (EHP) exhibit a similar syndrome as seen in fish: reduced growth rate and production [6,7,8].

EHP was discovered in 2004 and identified as a new species in 2009, which can infect *Penaeus monodon* and *Penaeus vannamei* [8,9,10]. Its *SSU rRNA* is 84% identical to that of *Enterocytozoon bieneusi,* which causes life-threatening diarrhea [8]. Mature EHP spores can spread directly between shrimps through the water and cause hepatopancreatic microsporidiosis (HPM), resulting in ultimately significant economic losses [11]. Presently, EHP has been widely spread in most parts of Asia and South America [12], and is considered to be the third major disease of shrimp, after white spot syndrome virus (WSSV) and acute hepatopancreatic necrosis disease (AHPND) [6]. Methods for EHP detection are mainly based on several conserved molecular markers, such as SSU rDNA, *SWP1*, *beta-tublin*, and *ptp2*, using loop-mediated isothermal amplification (LAMP), nested PCR, quantitative PCR, and a CRISPR-Cas12a fluorescent cleavage assay (RPA-Cas12a) [13,14,15,16,17,18,19,20,21,22,23,24,25]; however, protein targets are rarely used.

The spore wall of a mature spore of microsporidia (infective phase) consists of three layers: an electron-dense proteinaceous outer layer (exospore), an electron-transparent chitinous inner layer (endospore), and an underlying plasma membrane [26,27]. It facilitates the maintenance of both spore shape and constant internal pressure to resist harsh environmental conditions prior to germination and host infection [26,27,28]. Spore wall proteins (SWPs) are the major components of the spore wall, which directly interact with host cells during the infection and play important roles in adhesion to host cells, ion channels, energy transfer, signal transduction, and enzymatic reactions [28,29,30]. Several SWPs have been characterized that may be involved in host adhesion, including EcSWP1, EcEnP1, EcEnP2, EcSWP3, and EcCDA from *Encephalitozoon cuniculi,* and EiSWP1, EiSWP2, and EiEnP1 from *E. intestinalis* [30,31,32,33,34,35]. To date, 14 hypothetical SWPs have been identified from *N. bombycis* by proteomic analysis [36]. Among them, eight SWPs were localized using an indirect immunofluorescence assay (IFA) and immunoelectron microscopy (IEM), including NbSWP30(SWP1), NbSWP25(SWP2), NbSWP32(SWP3), NbSWP5, NbSWP7, NbSWP9, NbSWP11, and NbSWP12 [36,37,38,39,40,41]. NbSWP26 participates in host cell adhesion and infection through its interaction with the host protein Bmtutl-519 [42,43,44]. The AlocSWP2 in *Paranosema (Antospora)* *locustae* has high levels of amino acid sequence identity with its homologue protein in *Encephalitozoonidae* and *Nosema*. Further analysis showed that AlocSWP2 was localized in the wall of sporoblasts, sporonts, and meronts, and may play a crucial role in the infection of locusts [45]. Amino acid sequence analysis revealed that EhSWP1 contains three heparin-binding motifs (HBMs), which may be involved in the binding of EHP spores to heparin on the surface of host cells [46]. In addition, previous studies pointed out that EhSWP2 (also named EhSWP26), EhSWP7, EhSWP12, and EhEnP1 are potential targets for EHP detection [47,48]. However, there is still a lack of comprehensive understanding of SWPs and the determination of effective protein targets for the immunodetection of EHP.

In this study, EHP spore surface proteins were isolated with the improved SDS method. High-abundance surface proteins (HASPs) were identified through LC-MS/MS, and bioinformatics analysis was performed based on EHP genome data [49]. Interestingly, a novel spore wall protein, named as EhSWP3, was identified with an indirect immunofluorescence assay (IFA) and immunoelectron microscopy (IEM). Further analysis showed that EhSWP3 has good species specificity and high sensitivity, making it a suitable target for EHP detection.

## 2. Materials and Methods

### 2.1. Purification of EHP Spores and Extraction of Spore Wall Proteins

In October 2018, EHP-infected *Penaeus vannamei* specimens (weight 8 to 15 g) were collected from commercial shrimp ponds in Fujian, China. The purification of EHP spores was performed according to a previously described method, with some modifications [49]. Briefly, the infected hepatopancreas was dissected and homogenized with an electric tissue grinder. Tissue debris were removed by filtering the product twice through a syringe containing 3 layers of absorbent cotton, followed by two rounds of sucrose density gradient centrifugation (30%, 45%, 60%) (*m*/*m*) at 40,000× g for 30 min each. The fraction with 60% sucrose concentration was collected and rinsed to obtain purified EHP spores, and was stored at 4 °C for later use.

Surface proteins were extracted following the procedure of Southern TR. et al. [36]. Briefly, spores (10^7^ cells) were suspended in 200 µL extract buffer (1% SDS, 10% sucrose, 5% β-mercaptoethanol, 60 mmol/L Tris, 100 mmol/L EDTA, 250 μM HgCl_2_), incubated at 60 °C for 30 min, centrifuged at 12,000× *g*, 4 °C for 10 min, and the supernatant containing surface proteins was collected for later use. Spores in control groups were suspended using 0.01 M PBS buffer (pH 7.4). Then, 20 µL supernatant was subjected to 12% SDS-PAGE analysis and silver nitrate staining.

To detect the presence of nuclear proteins and cytoplasmic proteins in the extracted proteins, the pellet generated by the centrifugation was resuspended in 0.01 M PBS (pH 7.4) and used for staining [50]. Briefly, fluorescent brightener 28 (Sigma, St. Louis, MO, USA) and propidium iodide (PI) were diluted into 1 µg/mL with 0.01 M PBS (pH 7.4). Fluorescent brightener 28 was used for the staining of chitin on the spore surface, while PI (Sigma) was used for EHP spore nucleus staining. Then, 10 µL resuspension was mixed with 50 µL dual-color staining solution, and incubated at room temperature for 5 min. Then, 10 µL mixture was added on the slides coated by polylysine and checked under a fluorescence microscope (OLYMPUS, Tokyo, Japan).

**Figure 1:**
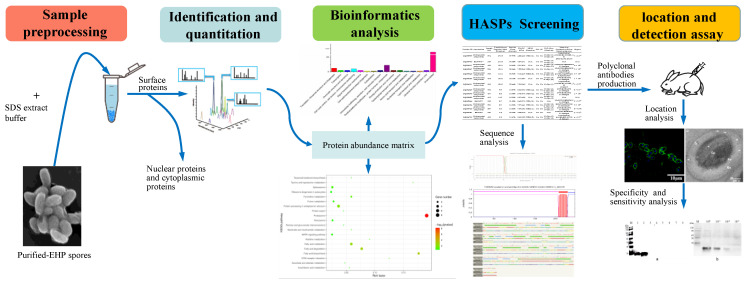
Flow chart of surface proteins analysis, identification, and characterization of EhSWP3. Surface proteins were extracted using the improved SDS method from purified EHP spores, inactivated by 250 μM HgCl2, and then were subjected to LC-MS/MS and bioinformatic analysis. Proteins with unique peptides ≥5 and coverage ≥10% were identified as high-abundance surface proteins (HASPs). Polyclonal antibodies against EhSWP3 (PAbs anti-EhSWP3) were produced by immunized rabbits using the recombinant EhSWP3 and validated by indirect immunofluorescence assay and immunoelectron microscopy. Then, the specificity and sensitivity analysis of PAbs anti-EhSWP3 for EHP was performed.

The procedures of surface protein extraction and analysis are shown in Figure 1.

### 2.2. LC-MS/MS Analysis and Protein Identification

The supernatant containing the surface proteins was condensed in a freeze dryer for 24 h, and then digested by trypsin (trypsin: protein = 1:40) at 37 °C for 16 h. The digested mixture was extracted with 0.1% trifluoroacetic acid in 60% ACN three times. The extracts were pooled, dried by a vacuum centrifuge, and subjected to liquid chromatography analysis on Zorbax 300SB-C18 peptide Traps (Agilent Technologies, Santa Clara, CA, USA) using an RP-C18 column (0.15 mm × 150 mm) (Column Technology Inc., Fremont, CA, USA) with solvent A (0.1% formic acid) and buffer B (0.1% formic acid in acetonitrile, 84% ACN). After the column was equilibrated with 95% buffer A, the sample was loaded and subjected to gradient elution with the following parameters: 4–50% buffer B for 50 min, 50–100% buffer B for 4 min, and 100% buffer B for 6 min. Then, the separated proteins were detected by a QExactive mass spectrometer (Thermo Fisher, Waltham, MA, USA) in full scan with a positive polarity and subsequent data-dependent acquisition (DDA) model.

The raw mass spectrometry data were analyzed using Mascot 2.2 against the *E. hepatopenaei* protein database deposited in Uniprot (https://www.uniprot.org/; accessed on 15 June 2020) with the following parameters: trypsin with a maximum of two missed cleavages, carbamidomethylation, methionine oxidation, reverse decoy mass tolerance 20 ppm, error tolerance 0.1 Da, and a mascot score of 20 or greater. Peptides were excluded for further analysis when the matching *p ≤* 0.05. A protein was considered when at least two different peptides were identified. Proteins that were unambiguously identified as nuclear and cytoplasmic ones were filtered out from raw proteins data by expert annotation information.

### 2.3. Bioinformatics Analysis

Proteins were classified via gene ontology (GO) annotation based on three categories: cellular component (CC), biological process (BP), and molecular function (MF). COG and KEGG were analyzed using WebMGA (http://weizhong-lab.ucsd.edu/webMGA/server/cog/; accessed on 5 August 2020) and KAAS (https://www.genome.jp/tools/kaas/; accessed on 5 August 2020), respectively. To investigate the biological significance of annotated proteins, Omicsbean (http://www.omicsbean.cn; accessed on 5 August 2020) was used to analyze the functional features based on enriched GO terms and KEGG pathways. Pathway enrichment was analyzed using Fisher’s exact test and visualized using ggplot2 v3.3.0. Uncharacterized proteins or SWPs with a unique peptide count ≥5 and coverage ≥10% were considered to be high-abundance surface proteins (HASPs). Conserved domains were predicted with PFAM (http://pfam.janelia.org/; accessed on 5 August 2020). Signal peptide sequences and transmembrane domains were predicted using SignalP 5.0 (http://www.cbs.dtu.dk/services/SignalP/; accessed on 5 August 2020) and TMHMM 2.0 (http://www.cbs.dtu.dk/services/TMHMM-2.0/; accessed on 5 August 2020), respectively. Putative N- and O-glycosylation sites were analyzed with NETNGLYC (http://www. cbs.dtu.dk/services/NetNGlyc/; accessed on 5 August 2020) and NETOGLYC (http://www.cbs.dtu.dk/services/NetOGlyc/; accessed on 5 August 2020), respectively. The heparin-binding motifs (HBMs) were predicted with PatScanUI (https://patscan.secondarymetabolites.org/; accessed on 5 August 2020). Sequence similarity searches were performed by BLASTP against the NCBI database with an E-value cutoff of 1.0 × 10^3^. Finally, 2D topology was predicted by Phyre2 (http://www.sbg.bio.ic.ac.uk/phyre2; accessed on 5 August 2020).

### 2.4. Gene Cloning and Recombinant Protein Expression of EhSWP3

PCR primers with restriction enzyme sites were designed based on the genome DNA sequence of *EhSWP3* (GenBank No. OQS55745) using Primer 6.0. The forward primer *EhSWP3-F*-*Bam*HI (5′-CGCCGGATCCATGTTATTTTCTACAATATTATT-3) and reverse primer *EhSWP3-R*-*Sal*I (5′-CGCGTCGACAGGTTGCATTGAATC ATCTGCATTT-3′) were used for PCR amplification in a 25 μL reaction system with Ex Taq DNA polymerase (TaKaRa). The reaction conditions were as follows: 95 °C for 5 min, followed by 30 cycles of 95 °C for 30 s, 59 °C for 30 s, and 72 °C for 60 s, and a final extension at 72 °C for 10 min. The amplified fragments (747 bp) were cloned into the expression vector pET28a and transformed into *Escherichia coli* Roseta (DE3). A single transformed colony was grown in 5 mL LB medium containing 50 μg/mL Kanamycin overnight at 37 °C with shaking at 180 rpm. Then, IPTG was added to a final concentration of 0.5 mmol/L to induce protein expression at 37 °C for 12 h. Cells were obtained via centrifugation at 12,000× *g* and 4 °C for 10 min, followed by ultrasonic treatment in ice for 10 min. The supernatant and precipitation were collected separately and analyzed by 12% SDS-PAGE electrophoresis. The extracted proteins were then purified with Ni-IDA-Sepharose Cl-6B affinity chromatography (Novagen, Madison, WI, USA) for later use.

### 2.5. Polyclonal Antibodies Preparation and Western Blotting Analysis

The preparation of polyclonal antibodies against EhSWP3 (PAbs anti-EhSWP3) was performed as follows: purified recombinant EhSWP3 was intradermally injected into New Zealand white female rabbits (2–2.5 kg) at a dose of 0.4 mg protein per rabbit, and 0.01 M PBS (pH 7.4) buffer was used as the control. One week after three booster injections, rabbit blood was collected, and serum containing PAbs anti-EhSWP3 was obtained via centrifugation and purified to a concentration of 1 mg/uL, and stored at −20 °C for later use.

For Western blot analysis, the purified recombinant rEhSWP3 was separated by 12.5% SDS-PAGE and then transferred onto a PVDF membrane (0.22 mm). The membrane was blocked with 5% nonfat dry milk in TBST buffer (150 mM NaCl, 20 mM Tris-HCl, 0.05% Tween-20) at 37 °C for 60 min, then incubated overnight with the rabbit PAbs anti-EhSWP3 (1:2000) at 4 °C. Normal rabbit serum was used as the control. After three washes with TBST buffer (10 min for each time), the membrane was incubated with goat anti-rabbit HRP-labeled antibodies (1:5000) at 37 °C for 60 min, followed by a final wash with TBST buffer. Bands were visualized using an enhanced chemiluminescence (ECL) detection system (Peiqing Chemiluminescence imaging System JS-1070EV, Shanghai, China).

### 2.6. Indirect Immunofluorescence Assay (IFA)

In the indirect immunofluorescence assay (IFA), purified EHP spores were placed on slides coated with 0.01% polylysine, fixed with 4% paraformaldehyde at room temperature for 15 min, and permeabilized with 2% Triton X-100 for 15 min, which was followed by three washes with 0.01 M PBS (pH 7.4). Subsequently, slides were blocked with 10% goat serum+ 5% BSA at room temperature for 60 min, and then incubated overnight with PAbs anti-EhSWP3 (1:100) at 4 °C. Normal rabbit serum was used as the control. After the third wash with 0.01 M PBS (pH 7.4), goat anti-rabbit IgG (1:100) conjugated with the fluorescein isothiocyanate (FITC, Thermo Fisher Scientific) was added and incubated at 37 °C for 60 min. Each slide was stained with 30 µL 4, 6-diamidino-2-phenylindole dihydrochloride (DAPI, diluted to 1:1000, Thermo Fisher Scientific) to label the nucleus for 15 min at room temperature, sealed with resin, and visualized using a confocal laser scanning microscope (OLYMPUS, Japan).

### 2.7. Immunoelectron Microscopy (IEM) Analysis

Purified EHP spores were fixed overnight (4% paraformaldehyde + 0.1% glutaraldehyde + 4% sucrose + 0.1 M sodium cacodylate in 0.01 M PBS (pH 7.4)) and washed with 0.1 M sodium cacodylate 3 times. Then, spores were resuspended in 0.01 M PBS (pH 7.4) containing 0.1% glycine, 4% sucrose, and 0.1 M sodium cacodylate at 4 °C for 30 min. Then, 1% agarose was added, followed by centrifugation at 12,000× *g* for 10 min, with the product then cut into uniform pieces (1 cm^3^). The pieces containing purified EHP spores were dehydrated through a gradient methanol (30%, 50%, 70%, 80%, 95%, and 100%), infiltrated in K4M resin overnight, and then permeabilized under the ultraviolet irradiation at −20 °C for 48 h. Ultrathin sections were prepared with an ultramicrotome (LEICA, Wetzlar, Germany) and placed on nickel grids for immunostaining. The grids were blocked in 1% BSA-5% normal goat serum in 0.01 M PBS for 1 h at room temperature, incubated with PAbs anti-EhSWP3 (1:25) for 1 h, and washed six times with 0.01 M PBS (pH 7.4). Normal rabbit serum was used as the control. Subsequently, the grids were incubated with gold-conjugated goat anti-rabbit IgG (1:50) (Jackon ImmunoResearch, West Grove, PA, USA) for 1 h and washed with ddH_2_O six times. Finally, the grids were stained with 1% uranyl acetate solution for 10 min and 1% lead citrate solution for 5 min, which was followed by three washes with ddH_2_O. The grids were visualized with a transmission electron microscope (HITACHI, Tokyo, Japan) at an accelerating voltage of 90 kV.

### 2.8. Specificity and Sensitivity of Ployclonal Antibodies

To detect the specificity of PAbs anti-EhSWP3, hepatopancreases (~30 mg) were obtained from five EHP-infected shrimps, homogenized, and incubated with lysis buffer (7.3% SDS, 10% sucrose, 5% β-mercaptoethanol, 60 mM Tris-HCl, 100 mM EDTA, and 2 μM PMSF) at 60 °C for 30 min. The homogenate was centrifuged at 12,000× *g* and 4 °C for 10 min. The supernatant was collected for later use. At the same time, the protein samples extracted from the spores of *Hepatospora eriocheir*, *Ameson portunus*, *Nosema bombycis*, *Vairimorpha (Nosema) ceranae*, *Pichia pastoris*, and the cells of *Staphylococcus aureus* were used as controls.

For the sensitivity analysis, the amount of EHP in the infected hepatopancreases was analyzed by PTP2-qPCR [20]. Briefly, fresh hepatopancreas tissue (~30 mg) from five EHP-infected shrimps was mixed with 500 µL CTAB extraction buffer (CATB 4 g, NaCl 16.34 g, 1 M Tris-HCl (pH 8.0) 20 mL, 0.5 M EDTA 8 mL, sterilized water up to 200 mL) and 20 µL proteinase K (20 mg/mL), and then incubated at 56 °C for 1 h. Finally, genomic DNA was isolated from the lysate using the phenol–chloroform method [14], precipitated by cold ethanol and solubilized in 80 µL ddH_2_O (final concentration 50 ng/µL). Specific primers were designed based on the nucleotide sequence of the *EHP-PTP2 gene* (GenBank No. MT249228): EHP-PTP2-F (5′ GCAGCACTCAAGGAATGGC3′) and EHP-PTP2-R (5′ TTTCGTTAGGCTT ACCCTGTGA 3′). qPCR amplification was performed in a 10 µL reaction system, including 1 µL DNA template (50 ng), 5 µL 2 × Hieff^®^ qPCR SYBR Green Master Mix (Yeasen, Shanghai, China), and 0.2 µM each of primer and appropriate ddH_2_O. The cycling parameters were as follows: 95 °C for 5 min, followed by 40 cycles of 95 °C for 10 s and 60 °C for 30 s in the LightCycler^®^ 96 (Roche, Indianapolis, IN, USA). All amplification products were detected by 1.5% agarose gel electrophoresis, followed by data analysis using the LightCycler^®^ 96 Software 1.1 (Roche).

In addition, total proteins from the hepatopancreases were serially diluted in 0.01 M PBS (pH 7.4) (10^−1^, 10^−2^ and 10^−3^) according to the quantified EHP loads from the PTP2-qPCR. Western blotting was performed as described above to detect the sensitivity of PAbs anti-EhSWP3. The band density was quantified using Image J software (https://imagej.net/software/fiji/downloads (accessed on 22 September 2021)) and analyzed using the drawing tools of Microsoft Excel. The EHP loads in the hepatopancreases were calculated using the following formula: copies/mg = (copies/µL) × (80 µL) × (30 mg)^−1^.

## 3. Results

### 3.1. Character of the Surface Proteins of EHP Spores

**Figure 2:**
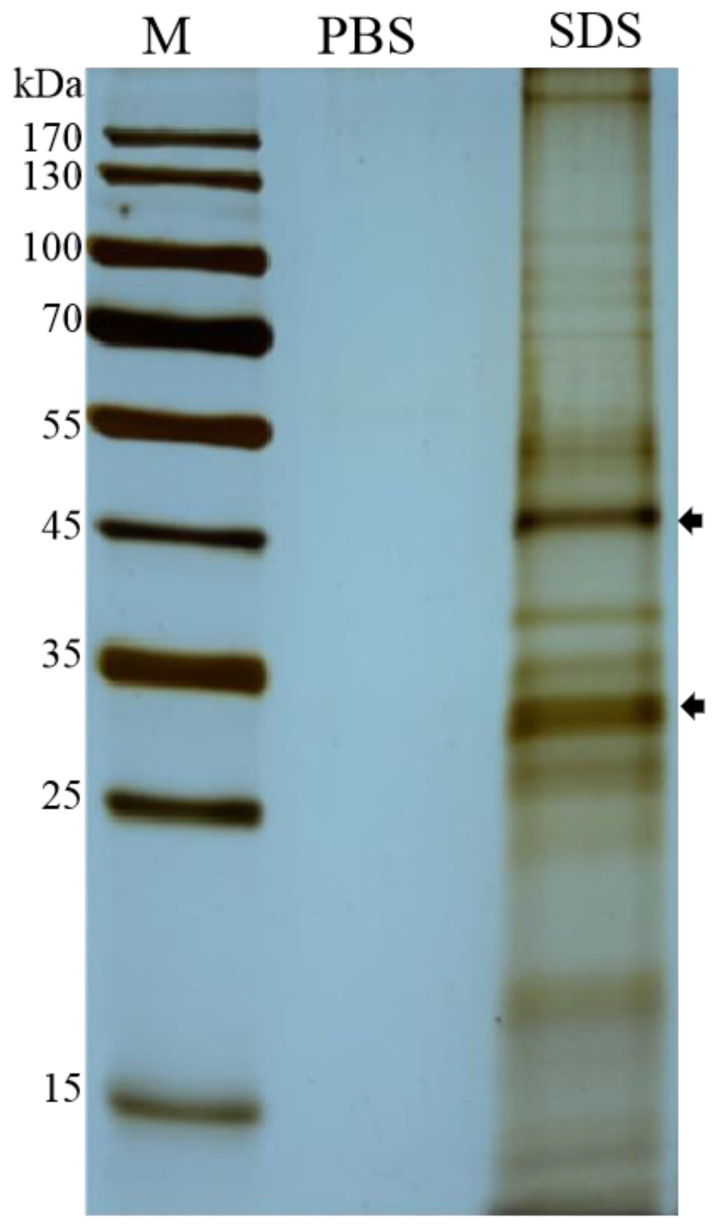
SDS-PAGE analysis of surface proteins of *Enterocytozoon hepatopenaei* spores. Lane M: protein maker; lane PBS: proteins extracted with 0.01 M PBS (pH 7.4); lane SDS: proteins extracted with 1% SDS.

The surface proteins extracted with 1% SDS or 0.01 M PBS (pH 7.4) were analyzed via SDS-PAGE. A variety of proteins with a relative molecular weight (MW) of 27 to 55 kDa were detected, including two major bands with the molecular weight of 32 and 48 kDa (Figure 2).

**Figure 3:**
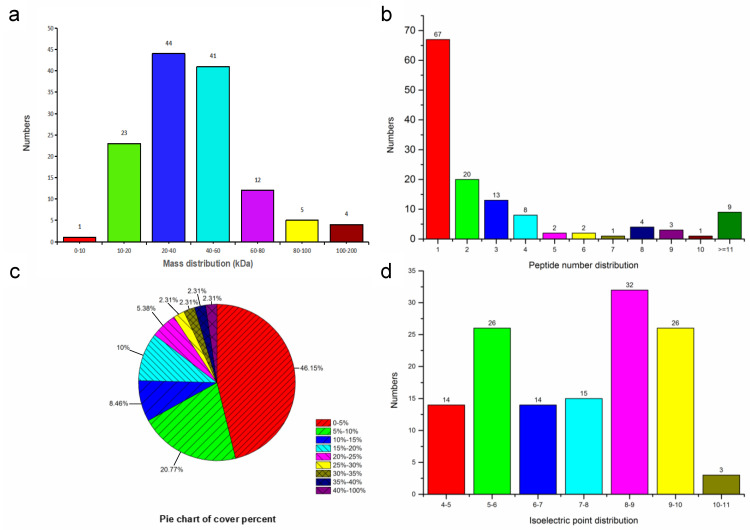
Overview of mass spectrometry data of 130 surface proteins extracted from *Enterocytozoon hepatopenaei* spores. (**a**) Molecular weight distribution. (**b**) Peptide number. (**c**) Peptide coverage (percent). (**d**) Statistical result of isoelectric point.

Based on LC MS/MS data, this study yielded 427 peptides and 130 proteins, including 71 uncharacterized proteins (Table 1 and Table S1). The 62 (47.69%) annotated proteins were enriched into GO cellular component categories, and 32 (24.62%) proteins were mapped to KEGG pathways (Table 1). Each of the 63 proteins (48.46%) contained more than two peptides. The coverage of 43 proteins (31.08%) exceeded 10% (Figure 3).

**Figure 4:**
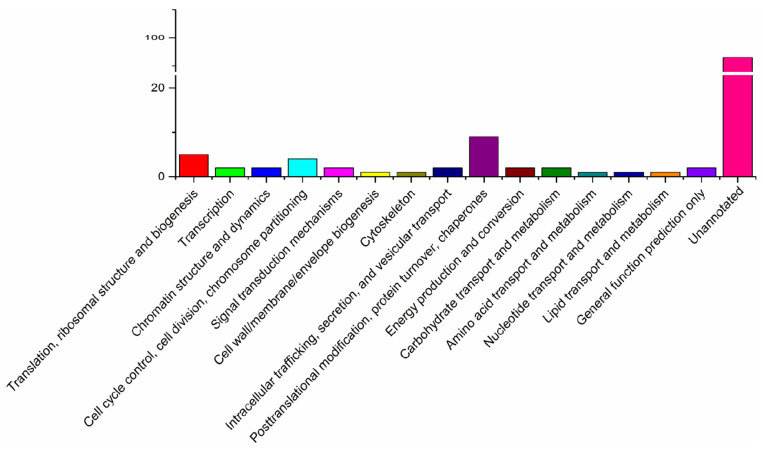
COG annotation of surface proteins of *Enterocytozoon hepatopenaei* spores.

The COG analysis revealed that the majority of abundant proteins were associated with post-translational modification, protein turnover, chaperons, signal transduction, cell wall/membrane/envelope biogenesis, energy production and conversion, lipid transport, and metabolism. In addition, 93 proteins were assigned to the unannotated category (Figure 4). These findings were consistent with the results of the GO analysis, whereby 62 proteins were enriched in a “cellular component”, seven proteins were enriched in an “extra cellular region part”, and seven proteins were associated with membrane-enclosed lumen, which were located on the cell surface and considered to be potential SWPs of microsporidia.

**Figure 5:**
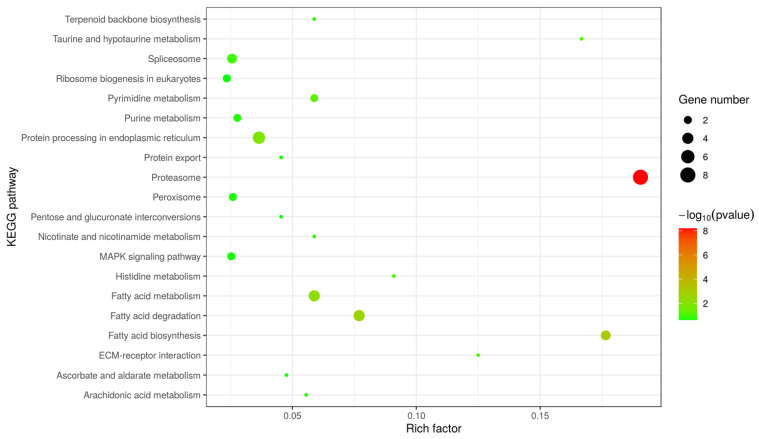
Top 20 KEGG pathways enriched from 130 surface proteins. Twenty KEGG pathways were obviously enriched from surface proteins of EHP spores (*p*-value of Fisher’s exact test < 0.01). MAPK: the mitogen-activated protein kinase; ECM-receptor: the extracellular matrix receptor. pvalue: *p*-value.

The results of the KEGG pathway enrichment analysis showed that 130 proteins were mainly enriched in 20 pathways, such as protein degradation (eight proteins) (*p* < 1.84 × 10^−9^) and fatty acid degradation and fatty acid metabolism (15 proteins) (Figure 5). Interestingly, an uncharacterized protein (OQS54254) was enriched into “ECM-receptor interaction”, which potentially involved the interaction between parasites and hosts. In addition, a protein (OQS53980) was enriched into the category of “ubiquitin mediated proteolysis”.

A total of 15 high-abundance surface proteins (HASPs) were identified with the threshold of unique peptide count ≥5 and coverage percent ≥10% (Table 2). Among them, six proteins, including the newly identified EhSWP3, contain transmembrane domains. Only EhSWP7 has a signal peptide sequence. Prediction results showed that 11 proteins contain potential N- and O-glycosylation sites, but proteins OQS53967 and OQS54511 only contain N-glycosylation sites. The amino acid sequence analysis found that nine HASPs have conserved heparin-binding motifs (HBM) (Table 2). Interestingly, homologous proteins of 13 HASPs, including EhSWP1 and EhSWP7, were also identified as SWPs in other microsporidia species.

**Figure 6:**
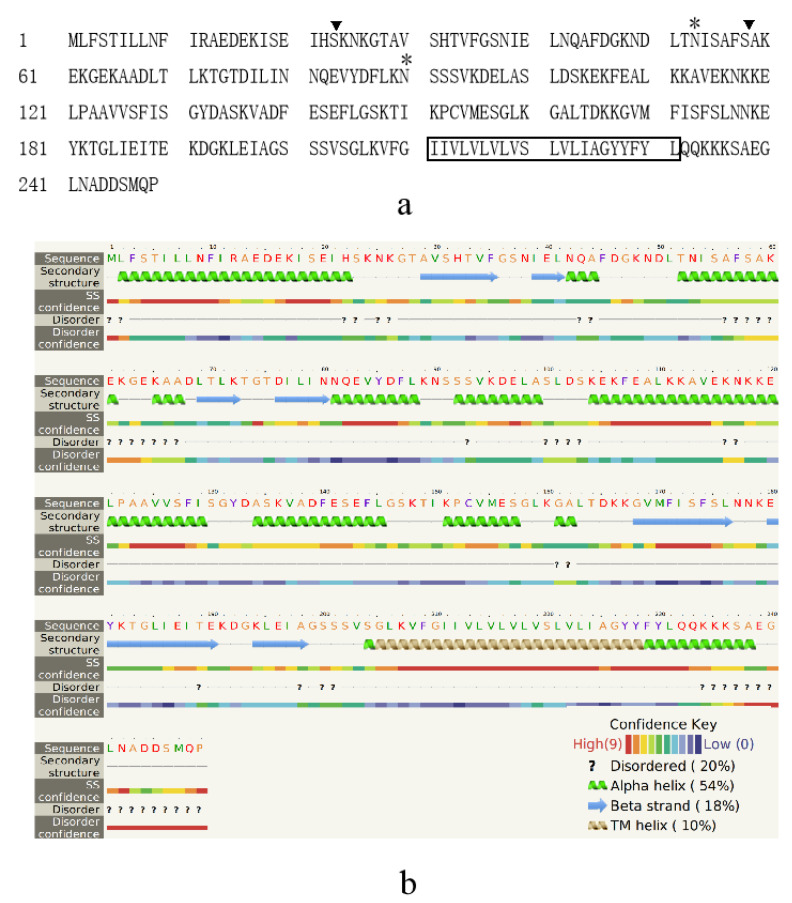
Amino acid sequence analysis and 2D structure prediction of EhSWP3. (**a**) Predicted post-translational modification sites. The transmembrane domain (TM) was boxed. N- and O-glycosylation sites were marked with “*” and “▼”, respectively. (**b**) The 2D structure of EhSWP3. The transmembrane domain (aa 209–231) was localized in the brown TM helix.

Of all the surface proteins, protein OQS55745 has 56.63% coverage and is the second highest abundance protein (21 peptide counts and 14 unique peptide counts). Because no homologue was found in GenBank, it was described as a new SWP and named as EhSWP3. Further analysis revealed that EhSWP3 is composed of 249 amino acid residues with a transmembrane domain, contained two N-glycosylation sites (AA^53^, AA^90^) and two O-glycosylation sites (AA^23^, AA^58^) (Figure 6a), and had a putative molecular weight of 27.1 kDa and an isoelectric point of 5.87 (Table 2). The 2D structure prediction showed that EhSWP3 is composed of more than eight α-helices and multiple coils, and the transmembrane domain is located at the C-terminus (amino acids 209–231) (Figure 6b).

### 3.2. Production of Polyclonal Antibodies against EhSWP3

**Figure 7:**
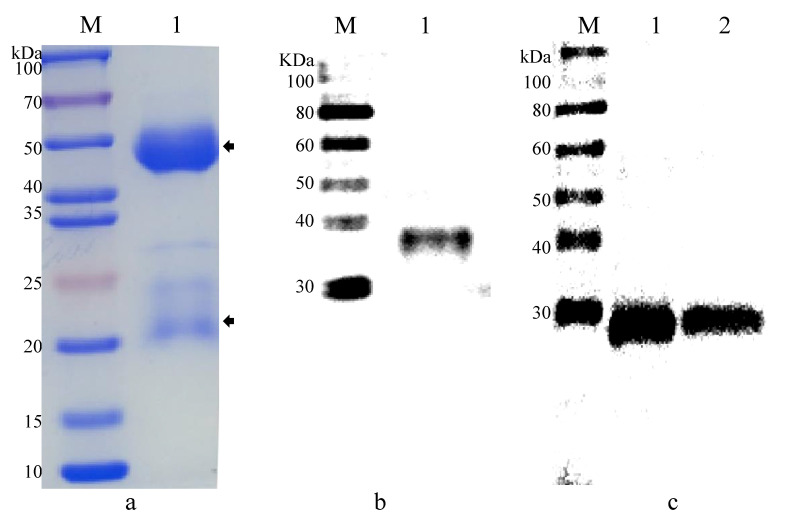
SDS-PAGE and Western blot analysis of PAbs anti-EhSWP3. M: protein molecular weight marker. (**a**) 1: The H-chain and L-chain of PAbs anti-EhSWP3 were marked with black arrows; (**b**) 1: purified recombinant protein rEhSWP3; (**c**) 1: total proteins extracted from EHP spores using SDS extraction method; (**c**) 2: total proteins extracted from EHP-infected hepatopancreas tissue of shrimp using SDS extraction method.

PAbs anti-EhSWP3 were produced by immunizing rabbits with recombinant EhSWP3 (rEhSWP3) and analyzed via SDS-PAGE. The rabbit PAbs anti-EhSWP3 consists of a 55 kDa heavy chain and a 25 kDa light chain (Figure 7a). In the Western blotting analysis, a single band (~27 kDa) was detected by PAbs anti-EhSWP3 from the total proteins of EHP spores and the EHP-infected hepatopancreas of shrimps. However, no specific bands were detected in the control group using normal rabbit serum. Therefore, the PAbs anti-EhSWP3 prepared in this study could specifically bind rEhSWP3 and EhSWP3.

### 3.3. Subcellular Localization of EhSWP3

**Figure 8:**
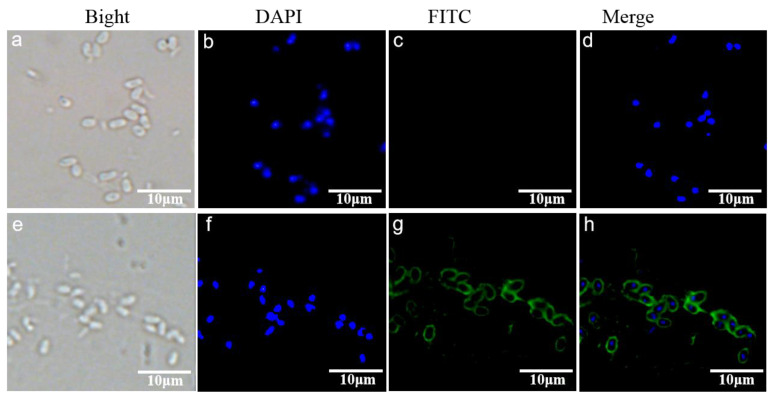
Immunofluorescence localization of EhSWP3 on the spore wall of purified *Enterocytozoon hepatopenaei* spores. Panel (**a**–**d**) normal rabbit serum. Panel (**e**–**h**): rabbit anti- EhSWP3 serum. (**d**) merged images of (**b**,**c**,**h**) merged images of (**f**,**g**). The blue fluorescence signal represents the nuclei of the spores.

An indirect immunofluorescence assay (IFA) was performed to localize EhSWP3 in mature EHP spores. The fluorescence signal was observed around the spore wall, while no signal was detected in the control group (Figure 8c,g), indicating that EhSWP3 is located on the surface of EHP spores, which is consistent with the transmembrane domain in its 2D structure prediction.

**Figure 9:**
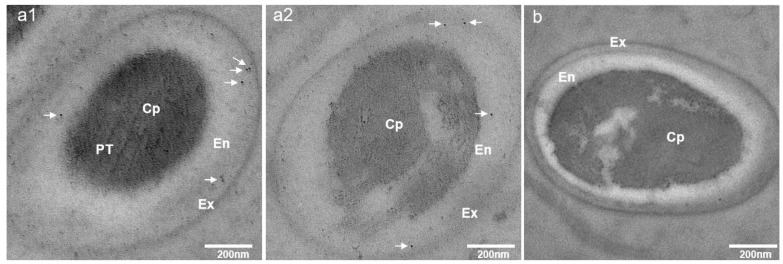
Immunoelectron microscopy localization of EhSWP3 on the spore wall of purified *Enterocytozoon hepatopenaei* spores. The sections were immuno-labeled with PAbs anti-EhSWP3 followed by GAR-IgG conjugated to gold (10 nm probe). (**a1**,**a2**) Electron micrographs of EhSWP3 in *E. hepatopenaei* spores. The presence of EhSWP3 was marked with white arrows. (**b**) The control was probed with normal rabbit serum. Abbreviations: Ex: exospore layer; En: endospore layer; Cp: cytoplasm; PT: polar tube.

To confirm the localization of EhSWP3, immunoelectron microscopy was performed. As shown in Figure 9a(1,2), signals were observed in the endospore of EHP spores, but scattered weak signals were detected in the cytoplasm. No signals were found in the control group (Figure 9b).

### 3.4. Specificity and Sensitivity of Polyclonal Antibodies Anti-EhSWP3

**Figure 10:**
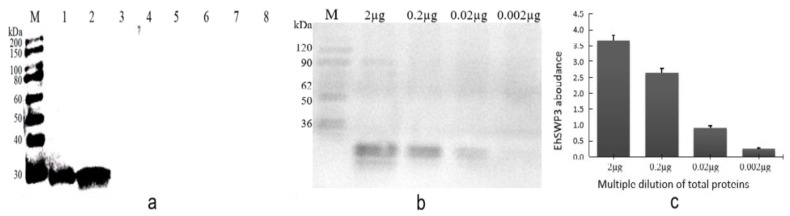
Specificity and sensitivity analysis of PAbs anti-EhSWP3. (**a**) Specificity analysis of PAbs anti-EhSWP3. M: protein marker, lane 1: proteins extracted from EHP spores, lane 2: proteins extracted from the hepatopancreas of EHP-infected shrimps, lanes 3–8: protein from the controls (*H. eriocheir*, *A. portunus*, *N. bombycis*, *V. ceranae*, *P. pastoris,* and *S. aureus,* respectively). (**b**) Sensitivity analysis of PAbs anti-EhSWP3. M: protein molecular weight marker. Here, 10 µL gradient dilution solution of total proteins extracted from shrimp hepatopancreases was loaded for each lane (lane 1: 2 µg, lane 2: 0.2 µg, lane 3: 0.02 µg, lane 4: 0.002 µg). (**c**) Gray analysis of Figure 10b.

The immunoblotting results showed that the PAbs anti-EhSWP3 prepared in this study could specifically bind to the target protein from the EHP spores and the hepatopancreases of EHP-infected shrimp, but no clear bands were detected in the control group (*H. eriocheir*, *A. portunus*, *N. bombycis*, *V. ceranae*, *P. pastoris*, and *S. aureus*) (Figure 10a), indicating that PAbs anti-EhSWP3 could specifically recognize EHP.

The PTP2-PCR results showed that the average loads of EHP in the hepatopancreases of EHP-infected shrimp was 2.7 × 10^4^ copies/mg. The immunoblotting results revealed that PAbs anti-EhSWP3 could detect EHP spores up to 10^−2^–10^−1^ (2.7 × 10^2^–2.7 × 10^3^ copies/mg) dilutions, but not lower (10^−3^) (Figure 10b,c). These results indicated that the minimum detection of EHP loads (2.7 × 10^2^ copies/mg) makes it a promising protein marker for the development of an immunodetection method for EHP detection.

## 4. Discussion

### 4.1. High-Abundance Surface Proteins (HASPs) of EHP

Spore wall proteins (SWP) play important roles in signal transduction, cell adhesion, ion transport, and parasite–host interactions by directly binding targets on host cell surface. A previous study identified 14 hypothetical SWPs from *N. bombycis*, using four protein extraction methods and proteomic analysis [36]. Subsequently, six more HSWPs (NbSWP25(SWP2), NbSWP5, NbSWP7, NbSWP9, NbSWP11, and NbSWP12) were confirmed as SWPs through subcellular localization analysis [37,38,39,40,41]. Therefore, species-specific SWPs were crucial for the formation of spore walls, and may also be involved in parasite–host interactions [51,52].

In this study, mature EHP spores were inactivated with 250 μM HgCl_2_, and then surface proteins were extracted using the improved SDS method. The inactivation of spores with HgCl_2_ can effectively avoid non-surface protein contamination due to spore germination. The concentration gradient experiment showed that a high concentration of SDS (e.g., 1.5%, 3.3%, 7.3%) can cause the rupture of EPH spores (>15%) (unpublished data). The release of sporoplasm could cause the contamination. Low concentrations of SDS (e.g., 0.5%, 0.1%) resulted in the rupture of a small number of EHP spores (<15%), but the protein extraction efficacy was low. In addition, the proteomic data were filtered to minimize the influence of other non-surface proteins. A total of 15 high-abundance surface proteins were identified via proteomic analysis, including EhSWP1 and SWP7. EhSWP1, the first EHP spore wall protein to be reported, was confirmed to be a virulence factor involves in host–parasite interactions by tethering spores to the heparin on the host cell surface [46]. SWP7 is a homologue of sporoplasm surface protein 1 of *E. hellem* (EhSSP1), which was identified as a novel sporoplasm surface protein, and could bind to host cell mitochondria via a host voltage-dependent anion channel protein (VDAC) associated with the final steps of invasion in the invasion synapse [53]. Among the 13 uncharacterized proteins, nine were identified as homologues of known SWPs. Therefore, it is feasible to extract SWPs from EHP with the improved SDS method. In-depth studies will be helpful to elucidate the function of HASPs in the formation of spore walls and the parasite–host interactions.

### 4.2. Novel Species-Specific Protein Marker EhSWP3

Presently, no accurate subcellular localization information for EhSWP2 (EhSWP26), EhSWP7, EhSWP12, and EhEnP1 was available [47,48]. The high-abundance surface proteins (HASPs) identified in this study might be important marker candidates for the identification of microsporidia. The preliminary localization analysis of the identified protein OQS55745 on the inner layer of the spore wall was consistent with the presence of the transmembrane domain. Due to the high identity of OQS53873 with the well-known spore wall protein EhSWP2 [48], it was considered to be the same protein. Hereafter, OQS55745 was designated as EhSWP3.

Although homologues of EhSWP1, EhSWP2, and EhSWP7 could be found in genera *Nosema* and *Encephalitozoon* [46], EhSWP3 did not show a detectable similarity to any known spore wall proteins of microsporidia and other species in the present GenBank database. It indicated that EhSWP3 is probably a species-specific protein. EhSWP3 was detected on the plasma membrane of EHP in the proliferative stage (unpublished data), which was consistent with the transmembrane domain prediction. In addition, the high expression level of EhSWP3 gene was also detected in the hepatopancreas of shrimp with high or low EHP infection (unpublished data). These results suggested that EhSWP3 might play an important role in the proliferation or maturation of spores. Putative glycosylation and phosphorylation sites in the amino acid sequence also implied the functions of EhSWP3 in parasite–host interactions. Of course, further studies on the exact function of EhSWP3 will be needed.

### 4.3. Promising Potential of Anti-EhSWP3 for EHP Detection

To detect EHP specifically, sensitively, conveniently, and quickly, researchers have developed a variety of detection methods, such as SSU-PCR, SWP-PCR, qPCR, LAMP, and CASE-RAE PCR [13,14,15,16,17,18,19,20,21,22,23,24,25]. However, these methods mainly use DNA as the detection target, which cannot avoid the tedious steps of DNA extraction and are not easy to apply in prawn farms.

Therefore, it is extremely necessary to study convenient and fast immunoassay methods based on antibodies. Presently, the monoclonal antibody against the EhSWP2 protein has been successfully used to detect shrimps infected by EHP [48]. Compared with SWP1 and SWP2, EhSWP3 is a highly abundant protein located in the endospore wall of mature EHP spores, and no homologous protein was found in the current GenBank database. Therefore, EhSWP3 is an ideal detection target. In this study, PAbs anti-EhSWP3 was prepared, and it possessed a high species specificity and sensitivity in the detection of EHP in the hepatopancreas of infected shrimps. Our results indicated that EhSWP3 has the potential to be developed into an immunochromatographic test strip. Meanwhile, we are exploring the preparation technology of EhSWP3 monoclonal antibodies and have carried out the development of test strips.

## 5. Conclusions

Surface proteins of EHP spores were extracted using the improved SDS method and analyzed via mass spectrometry. A bona fide spore wall protein and biomarker, EhSWP3, was identified from 15 high-abundance surface proteins, based on both proteomic analysis and on being successfully located on the endospore of EHP spores. The polyclonal antibodies of anti-EhSWP3 show high species specificity and good detection sensitivity, which make EhSWP3 an ideal protein marker for the development of rapid immunoassay tools for EHP detection in the future.

## Figures and Tables

**Table 1:** Overview of protein identification, annotation, and enrichment analysis.

Category	Protein Number
Peptides	427
Proteins	130
Uncharacterized proteins	71 (54.62%)
GO categories of biological process	67 (51.54%)
GO categories of cellular component	62 (47.69%)
GO categories of molecular function	71 (54.62%)
KEGG pathway	32 (24.62%)

**Table 2:** Profile of 15 high-abundance surface proteins of *Enterocytozoon hepatopenaei* spores.

Protein ID	Annotation	Length (Aa)	Total/Unique Peptide Count (Number)	Peptide Cover (Percent)	PI/MW (kDa)	HBM (Number)	TM	SP	N/O-Glyc (Number)	Homolog Protein/Accession Number/Species	Expect
OQS53969	Uncharacterized protein	374	27/15	37.97%	4.88/42.6	HBM (1)	No	No	N-Glyc (1) O-Glyc (3)	hypothetical protein/ECANGB1_151 *Enterospora canceri*	7 × 10^−^^18^
OQS55745	EhSWP3 ^※^	249	21/14	56.63%	5.87/27.1	N/A	Yes	No	N-Glyc (2) O-Glyc (2)	N/A	N/A
OQS54917	Uncharacterized protein	459	15/11	24.40%	5.01/50.6	N/A	Yes	No	N-Glyc (4) O-Glyc (10)	NbHSWP4/EF683104 *N. bombycis*	3 × 10^−^^5^
OQS53967	Uncharacterized protein	326	13/11	38.96%	7.33/36.8	HBM (1)	No	No	N-Glyc (1)	NbSWP32/EF683103 *N. bombycis*	3 × 10^−^^5^
OQS55648	Uncharacterized protein	356	11/11	30.90%	9.12/40.9	HBM (3),	Yes	No	N-Glyc (4) O-Glyc (12)	hypothetical protein/OQS55748.1 *E.hepatopenaei*	3 × 10^−^^5^
OQS54254	Uncharacterized protein	473	14/10	24.10%	4.11/53.7	N/A	No	No	N-Glyc (3) O-Glyc (29)	SWP/EOB14572 *N. bombycis*	1 × 10^−^^7^
OQS54511	Uncharacterized protein	299	13/10	32.44%	7.50/34.5	N/A	No	No	N-Glyc (1)	hypothetical protein/ECANGB1_595 *E.canceri*	1 × 10^−^^21^
OQS54349	Uncharacterized protein	592	9/9	15.37%	8.62/69.2	HBM (4)	No	No	N-Glyc (6) O-Glyc (36)	SWP/EOB14572 *N. bombycis*	3 × 10^−^^13^
OQS55378	Uncharacterized protein	407	8/8	18.43%	8.78/47.4	HBM (2)	No	No	N-Glyc (1) O-Glyc (15)	SWP/EOB14572 *N. bombycis*	2 × 10^−^^6^
OQS53833	Uncharacterized protein	412	10/7	18.20%	9.27/45.8	N/A	Yes	No	N-Glyc (1) O-Glyc (5)	N/A	N/A
OQS53864	EhSWP1	228	8/7	21.93%	8.45/27.0	HBM (1)	No	No	N/A	NbSWP12/EF683112 *N. bombycis*	2 × 10^−^^29^
OQS54094	Uncharacterized protein	390	8/7	16.92%	7.57/44.1	HBM (1)	No	No	N-Glyc (5) O-Glyc (12)	EiSWP2/AF355749 *E. intestinalis*	3 × 10^−^^5^
OQS55748	Uncharacterized protein	387	6/6	16.80%	7.59/45.0	HBM (1),	Yes	No	N-Glyc (5) O-Glyc (1)	SWP/EOB14572 *N. bombycis*	3 × 10^−^^6^
OQS55031	EhSWP7	250	7/5	20.00%	5.28/27.8	N/A	No	Yes	N/A	NbSWP7/EOB13707 *N. bombycis*	2 × 10^−2^^5^
OQS54755	Uncharacterized protein	478	5/5	10.67%	8.95/55.3	HBM (3),	Yes	No	N-Glyc (2) O-Glyc (5)	hypothetical protein/EBI_22790 *E. bieneusi*	4 × 10^−^^73^

Total/Unique Peptide Count: the number of total/unique peptide counts matched in LC-MS/MS; HBM: heparin-binding motif; TM: transmembrane domain; SP: signal peptide domain; N/O-Glyc: N- Glycosylation site or O- Glycosylation site; PI: isoelectric point; MW: molecular weight; ^※^ this study.

## Data Availability

The data presented in this study are available in Table S1.

## Supplementary Materials

The following supporting information can be downloaded at: https://www.mdpi.com/2076-2607/10/2/367/s1, Table S1: List of 130 surface proteins from spores of *Enterocytozoon hepatopenaei*.
